# Horizontal gene transfer of Chlamydia: Novel insights from tree reconciliation

**DOI:** 10.1371/journal.pone.0195139

**Published:** 2018-04-05

**Authors:** Hyaekang Kim, Woori Kwak, Sook Hee Yoon, Dae-Kyung Kang, Heebal Kim

**Affiliations:** 1 Department of Agricultural Biotechnology and Research Institute of Agriculture and Life Sciences, Seoul National University, Seoul, Republic of Korea; 2 C&K genomics, Seoul National University Research Park, Seoul, Republic of Korea; 3 Department of Animal Resources Science, Dankook University, Cheonan, Republic of Korea; University of the Pacific, UNITED STATES

## Abstract

Recent comparative genomics studies have suggested that horizontal gene transfer (HGT) is one of the major processes in bacterial evolution. In this study, HGT events of 64 *Chlamydia* strains were investigated based on the pipeline employed in HGTree database constructed in our recent study. Tree reconciliation method was applied in order to calculate feasible HGT events. Following initial detection and an evaluation procedure, evidence of the HGT was identified in 548 gene families including 42 gene families transferred from outside of *Chlamydiae* phylum with high reliability. The donor species of inter-phylum HGT consists of 12 different bacterial and archaeal phyla, suggesting that *Chlamydia* might have even more various host range than in previous reports. In addition, each species of *Chlamydia* showed varying preference towards HGT, and genes engaged in HGT within *Chlamydia* and between other species showed different functional distribution. Also, examination of individual gene flows of niche-specific genes suggested that many of such genes are transferred mainly within *Chlamydia* genus. Our results uncovered novel features of HGT acting on *Chlamydia* genome evolution, and it would be also strong evidence that HGT is an ongoing process for intracellular pathogens. We expect that the results provide more insight into lineage- and niche-specific adaptations regarding their infectivity and pathogenicity.

## Introduction

*Chlamydiae* are a phylum of Gram-negative, obligate intracellular bacteria. They consist of four validly described groups (*Waddila*, *Parachlamydia*, *Simkania*, *and Chlamydiaceae* family) and five additional *Candidatus* families (*Criblamydiaceae*, *Clavichlamydiaceae*, *Piscichlamydiaceae*, *Parilichlamydiaceae*, and *Rhabdochlamydiaceae*) [1]. *Waddila*, *Parachlamydia*, and *Simkania* families have been detected as symbionts of protozoa and emerging pathogens causing infections in humans and animals, and *Chlamydiaceae* family includes 11 species capable of infecting mainly humans and a wide range of animal species [2, 3]. *Chlamydiaceae* family especially has significant impacts on human and animal health worldwide as it is successfully evolved to inhabit a wide spectrum of eukaryotic hosts, comprising from protozoa to placental mammals. The continuing discovery of new species infecting novel hosts and expanding host ranges of known species indicate importance of niche-specific gene acquisitions in *Chlamydia* [4].

Bacteria exchange their genetic information via horizontal gene transfer (HGT), and it has been considered to be one of a major driving force in the microbial evolution, with gene mutation [5, 6]. While accumulation of mutations is a slower process, HGT have a more substantial and immediate impact on the phenotypes of recipients [7]. Recent studies have also shown that horizontal acquisition of 'pathogenicity islands' directly change the virulent nature of many pathogenic bacteria [8]. HGT is known to occur via three modes: conjugation, transformation, phage-mediated transduction. The exact mechanism of HGT of *Chlamydia* is largely unknown, but previous laboratory works have reported that HGT and subsequent recombination can occur after co-infection within host body [9, 10]. Discovery of a number of phages has shown that phage-mediated transduction in *Chlamydia* is possible as well [11]. Traditionally, due to obligate intracellular nature and low chance of co-infections with more than one strain, however, HGT in *Chlamydia* had been considered unlikely. It has only recently become obvious that C*hlamydia* genomes contain full gene sets necessary for HGT [12]. Additional evidence for the occurrence of HGT was reported from studies on *C*. *trachomatis* strains in which it was discovered that they actively swap DNA even with strains infecting different parts of the body, and the recombination was not restricted to few “hotspots” [13]. Recent comparative studies detected traces of frequent HGT on several *C*. *psittaci* genomes as well [14].

Comparative genome analysis in many bacterial and archaeal studies have provided new insights into evolutionary history by exploring remnants of HGT events [15, 16]. In spite of their importance as global infectious diseases and increasing evidences of significant role of HGT, there has been only few exhaustive studies on gene transfer events in different *Chlamydia* species. In this study, we analyzed sets of homologous genes from 64 chlamydial strains from 8 different species and their homologs in 2,407 other prokaryotes using the pipeline employed in our previous study, HGTree database [17] to initially identify all putative HGT events that occurred within *Chlamydiae* and between *Chlamydiae* and non-chlamydial prokaryotes. HGTree is a comprehensive resource providing all feasible genome-wide HGT information for completely sequenced genomes of 2,472 prokaryotes (as of 17 March, 2015) by means of phylogenetic tree reconciliation method [17]. It is quite advantageous in inferring tree reconciliation-based HGT information with hundreds of organisms with a reasonable running time. Following initial identification of HGTs in *Chlamydia*e, in order to improve the accuracy of the identification, we additionally evaluated the reliability of each potential candidate HGT events and mapping assignments using RAxML [18] and RANGER-DTL 2.0 software package [19].

Tree reconciliation method is generally considered to be more powerful and sensitive in detecting HGT than distance-based methods [20], but it is computationally intensive and therefore its practical use has remained challenging for analysis of a large number of genomes [17]. Here, we provide extended insight into HGT in the evolution of *Chlamydia* by enabling this massive computation. We identified numerous feasible HGT events that has occurred in evolutionary lineages of *Chlamydia*. Donor organisms external to the *Chlamydiae* phylum were examined to have insights into the ecology of *Chlamydia*. We also examined gene flows of individual genes associated with chlamydia’s survival strategies in the host cell. We expect that the results presented in this work could explain how HGT have impact on niche- and lineage-specific evolution of *Chlamydia*.

## Materials and methods

### Chlamydial genomes used in this study

We used 64 completely sequenced chlamydial genomes representing 8 different species from the HGTree database for analysis (all available clinically isolated genomes of *Chlamydia abortus*, *Chlamydia pecorum*, *Chlamydia psittaci*, *Chlamydia caviae*, *Chlamydia pneumoniae*, *Chlamydia felis*, *Chlamydia trachomatis*, *Chlamydia muridarum*). The list of 64 analyzed strains is presented in the S1 Table.

### Identification of putative HGT events

In construction of HGTree [17], 2,472 completely sequenced prokaryotic genomes (156 Archaea and 2,316 Bacteria) were used. A total of 7,748,306 genes were scanned using HMMER (ver. 3.0) (E-value < 10–3) [21]. RNammer (ver.1.2) was applied to detect 16S rRNA sequences in each genome [22]. In order to predict homologous gene sets, Mestortho orthology detection algorithm (ver.2.0.) was used [23]. Multiple sequence alignments of homologous gene sets and 16S rRNA sequences from corresponding species in the homologs sets were performed using CLUSTAL Omega (ver.1.2.1) [24]. Finally, RANGER-DTL-U software (ver.1.0) was employed to calculate all putative HGT events by reconciliation of the generated gene trees to 16S rRNA species trees. All of the detected HGT events but HGTs between same species were deposited to the database [17]. In this study, an initial screening with HGTree was performed to identify all of the putative HGT events on 64 chalmydial strains, and the candidate HGTs were used for further analysis to evaluate reliability.

### Evaluation of putative HGTs

To obtain HGTs with high reliability, additional analysis was applied following preliminary HGT detection with HGTree. Using RAxML [18], we reconstructed gene trees and species trees of 701 candidate homologs sets with history of HGTs, obtained from HGTree. For each RAxML analysis, we executed 100 rapid bootstrap inferences using GTR+CAT model for 16S rRNA genes and PROTCATJTT model for homologous gene sets. For species trees, 16S rRNA genes only from corresponding species in each set were used. 18S rRNA sequence from *Saccharomyces cerevisiae* was used in each species tree to root, and the sequence was removed using the Newick Utility (ver. 1.6) [25] after the species trees were generated. To evaluate candidate HGTs RANGER-DTL 2.0 [19] was applied. In contrast to RANGER-DTL 1.0, RANGER-DTL 2.0 is capable of sampling the space of all optimal reconciliations uniformly at random and computing multiple optimal reconciliations and accounting for the variability in optimal reconciliation scenarios [19]. Furthermore, to our knowledge, RANGER-DTL 2.0 is by far the only HGT detection program based on the tree reconciliation method that compute support values (SV) for individual DTL event inferences and species mapping assignments. After additional HGT detection with RANGER-DTL 2.0, total 3,004 HGT events in 669 genes were detected, and the average support value were 0.6463. In this study, we used support value cutoff of 0.9 to rule out ambiguous HGTs, and 1,030 HGTs in 548 genes were remained.

### Functional category assignments of transferred genes

Detected genes were assigned to the NCBI clusters of orthologous group (COG) catalogue [26]. COGnizer software was used to examine in which The Kyoto Encyclopedia of Genes and Genomes (KEGG) pathways the transferred genes are associated [27].

## Results & discussion

### Analysis of donor lineages in HGT events

**Fig 1:**
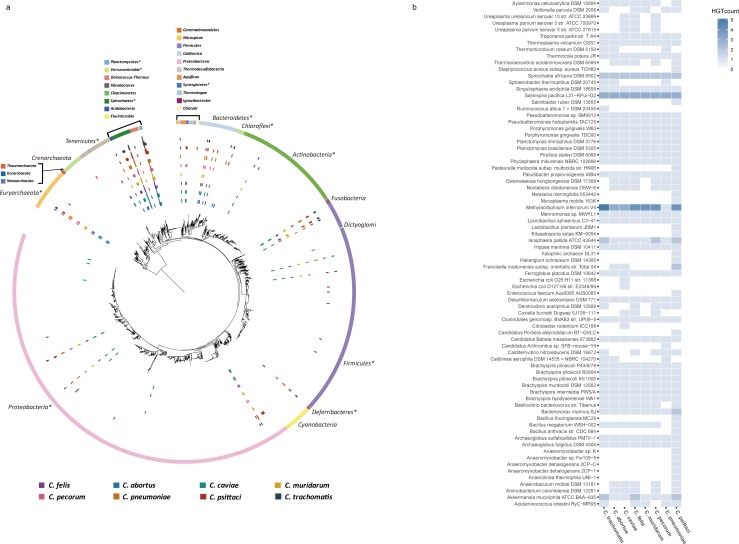
Donor organisms of HGT of 8 *Chlamydia* species. (A) This figure illustrates a global pattern of HGT in 8 *Chlamydia* species analyzed. The phylogenetic tree of 2,472 prokaryotic species was constructed with FastTree (ver. 2.9) based on multiple sequence alignment of 16S rRNA. Tree was visualized using Interactive Tree of Life Version 3.4.3 (http://itol.embl.de/) [28]. Each colored strip of the outer circle represents different phylum of bacteria and archaea. The actual donor phylums are highlighted with asterisk. From inside out, circled bar charts represent *C*. *abortus*, *C*. *caviae*, *C*. *felis*, *C*. *muridarum*, *C*. *pecorum*, *C*. *pneumoniae*, *C*. *psittaci*, and *C*. *trachomatis*, respectively. Each bar chart shows the gene transfers from corresponding organism in the tree, and the vertical bars represent the number of HGT events between corresponding donors and recipient organisms. (B) Heatmap showing the number of HGTs between *Chlamydia* and non-chlamydial donor species. Rows represent all identified donor species (SV ≥ 0.9); Columns represent recipient *Chlamydia* species. Only HGT events have SV ≥ 0.9 are shown here.

To investigate donor organisms of HGTs of 64 chlamydia strains, we inferred transfer events by reconciling each gene tree of 1,030 homologous gene sets to 2,472 completely sequenced prokaryotic species phylogenies. Following initial identification and evaluation procedure, we detected 1,030 highly reliable HGT events occurred between different species including *Chlamydia* for every gene family. It appeared that HGT has favorably occurred between species in the *Chlamydiae* phylum, but there was also a number of genes derived from organisms outside of the phylum. These organisms belong to 59 different genera, covering 12 bacterial and archaeal phyla which include a wide spectrum of life style (Fig 1). Numerous HGT events were subjected to occur between *Chlamydia* and *Proteobacteria*, *Spirochaetes*, whereas *Chlamydia* barely received genes from organisms in *Actinobacteria* phylum. Various species-specific trends of HGT were also observed in each chlamydial species. For example, several species in phylum *Firmicutes* such as *Bacillus anthracis* CDC 684, *Enterococcus faecium* Aus 0085, and *Staphylococcus aureus* subsp. *aureus* TCH60 transferred genes only to *C*. *psittaci*, and absence of HGT to *C*. *muridarum* from several donor organisms in *Bacteroidetes* were observed (Fig 1).

Although many of donor organisms such as *Neisseria meningitidis* and *Treponema pedis* are known to be pathogenic to vertebrates or host associated, there exist other donor species which are typically considered as free-living bacteria including *Isophaera pallida* ATCC 43644, *Methylacidiphilum infernorum* V4, and *Salinispira pacifica* L21-RPU1-D2. First, for the donor species that occupy very similar niches as *Chlamydia*, many organisms residing and causing infections in respiratory system or urogenital organs in human or other animals were identified, in which most of the *Chlamydia* species are associated. Additionally, our result shows an occurrence of several HGTs with commensal organisms isolated from gastrointestinal (GI) tract of mammals. For example, ribose-phosphate pyrophosphokinase gene was transferred from *Ureaplasma parvum* which inhabit genital areas (SV: 1.0), and *Enterococcus faecium* transferred gene encoding ABC transporter family protein to *C*. *psittaci* (SV: 1.0). In previous study, it was discovered that not only *Chlamydia* can efficiently infect the GI tract of all hosts, including humans, but GI tract can also act as reservoir sites for chlamydial persistent infection [29, 30]. It has been reported that the GI tract is also considered to be a hot spot for HGT among bacteria [31].

In addition to bacteria residing in human or animal body sites, we observed a big proportion of environmental donor species which inhabit aquatic or terrestrial habitats (Fig 1). For instance, Lysyl-tRNA synthetase gene was transferred from aquatic bacteria, *Caldilinea aerophile* (SV: 0.97), and gene encoding MiaB-like tRNA modifying enzyme was transferred from *Thermanaerovibrio acidaminovorans* DSM 6589 residing terrestrial environments (SV: 1.0). The mechanisms by which gene transfer of *Chlamydia* occurring is still unclear. A number of phages required for transduction were discovered and DNA transfers were observed after host co-infection of multiple strains in many studies [9–11]. *Chlamydia*e phylum is considered as a group of successful parasites that have an extremely broad host range and distributed ubiquitous in nature. There have been only four families (*Parachlamydiaceae*, *Simkaniaceae*, *Waddliaceae*, *and Criblamydiaceae*) discovered within the phylum which can grow in natural hosts like amoebae [2]. However, it has been reported in many studies that a large number of rRNA sequence of chlamydia-like organisms are detected in various environmental samples such as soil, water, hot spring, and activated sludge samples [4, 32, 33]. Our results suggest that *Chlamydia* may have even more various host range than we have thought and potentially exchange genes in as yet unknown natural host. It also suggests the hypothesis that there may exist a novel gene transfer mechanism enabling *Chlamydia* to exchange genes with free-living organisms.

### Variation in the effect of HGT between species

**Fig 2:**
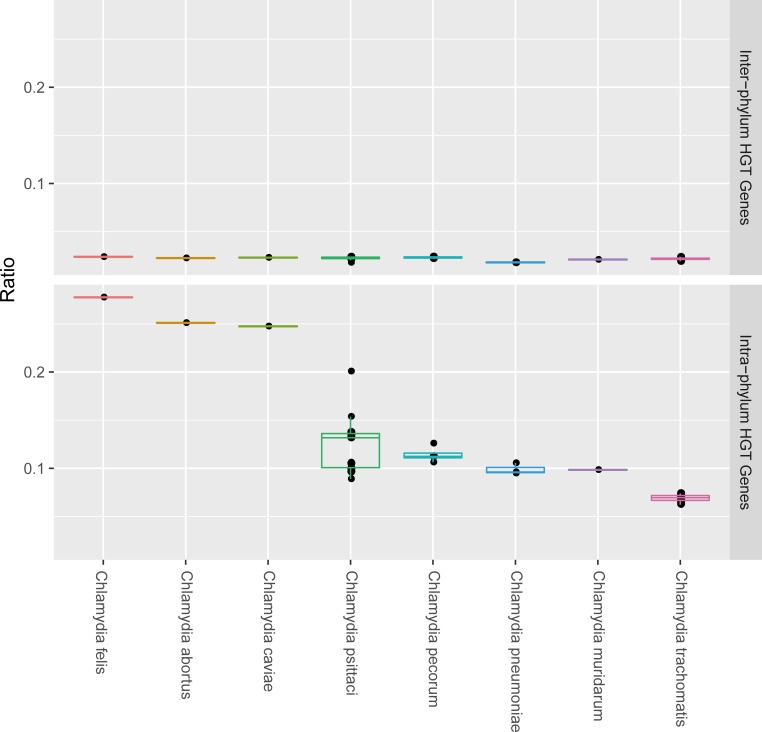
Distribution of the ratio of transferred genes of 64 chlamydial genomes. Boxplots show the distribution of the ratio of transferred genes for organisms in each *Chlamydia* species. Each plot shows the distribution of ratios of transferred genes from organisms outside of *Chlamydiae* phylum (Top) and ratios of transferred genes from *Chlamydia* (Bottom). The x-axis indicates of 8 *Chlamydia* species used in this study, and the y-axis indicates ratio of the number of transferred genes in the number of total gene in each strain. The great amount of variability in the number of transferred genes between species is derived mainly from intra-phylum transfer events, on the other hands, the ratio of the inter-phylum transferred genes is shown consistently low across all strains.

**Fig 3:**
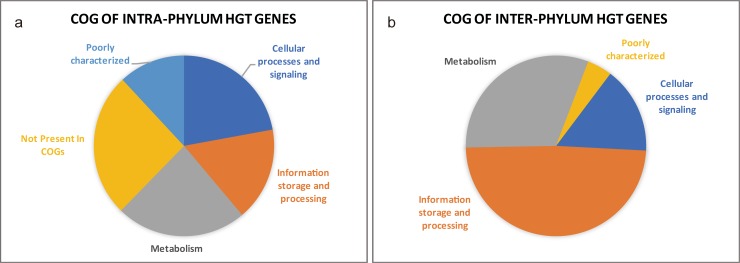
Distribution of received HGT gene sets across COG functional categories. The functional categories are according to the COG database [26]. (A) It shows the distribution of genes received from *Chlamydia*. (B) It shows the distribution of received genes from organisms other than *Chlamydiae* phylum. Genes from all functional categories (Cellular processes and signaling, Information storage and processing, and Metabolism) are subject to transfer. In contrast, in inter-phylum transfers, genes related to metabolism, and information storage and processing are assigned relatively high (31% and 49%, respectively), while only 16% of cellular processes and signaling genes are assigned.

Analysis of 64 chlamydial genomes using the HGTree database identified that 701 gene families were inferred to have undergone one or more HGT, with 97 gene families transferred from outside of *Chlamydiae*. Further examination of the 701 HGT-acquired genes with RAxML and RANGER-DTL 2.0 resulted in 548 putative genes including 42 non-Chlamydia originated genes with high reliability. The percentage of received genes was markedly variable across the species, being more than threefold greater in *C*. *felis* lineage (30.1% of total genes), *C*. *abortus* lineage (27.4% of total genes), and *C*. *caviae* lineage (27.1% of total genes) than in *C*. *trachomatis* (8.15~9.66%) (Fig 2). The great amount of variability in the number of transferred genes between species is derived mainly from intra-phylum transfer events, on the other hands, the ratio of the inter-phylum transferred genes is shown consistently low across all strains. We also looked at the distribution of all detected gene families across COG functional categories [26]. We found that genes from all functional categories (Metabolism, Information storage and processing, and Cellular processes and signaling) are subject to transfer (Fig 3). In contrast, in inter-phylum transfers, genes related to metabolism, and information storage and processing are assigned relatively high (31% and 49%, respectively), while only 16% of cellular processes and signaling genes are assigned (Fig 3). These results indicate that there may exist genetic barriers of inter-phylum HGT in chlamydial genomes. Gene transfer between distantly related organisms is known to occur less frequently than between closely related organisms since genetic mechanisms and different genome organization can act as constraints for inter-phylum HGT [34, 35].

As mentioned above, the *C*. *felis* and *C*. *abortus* genome displayed the highest ratio of HGT genes in all the species, with 30.1% and 27.4% of total genes, respectively. *C*. *abortus* is the most recently diverged species which mainly responsible for enzootic abortion in sheep and cattle [36]. There is a strong correlation between adaptation and HGT. It is known that bacteria can respond to SOS triggered by environmental stress and promote horizontal distribution of essential genes for survival [37]. In a recent study, 190 recombination events were observed in 12 *C*. *trachomatis* recombinants under antibiotic pressure [38]. It is also reported that mismatch repair gene deficient bacteria have significantly increased the rate of HGT and subsequent recombination of those genes[39]. In this way, HGT can be used to speed up the rates of adaptation in new environments [40], on the other hand, it is kept at a minimal level [41]. Therefore, high ratio of HGT genes of *C*. *abortus* can be explained as the result of ongoing adaptation to a new pathogenic lifestyle in placenta. In contrast, all of the *C*. *trachomatis* lineages show relatively low ratios, with an average of 9.08% of genes transferred. Possible explanation for this phenomenon is that *C*. *trachomatis* has very long evolutionary history since its divergence from the other *Chlamydia* approximately 6 million years ago [42], and unlike other *Chlamydia* that infect across multiple host species with histories of frequent host species jumps [14, 43], *C*. *trachomatis* has human beings as its exclusive natural host. Nevertheless, they are successfully adapted intracellular pathogens, which infections are among the most common of all human bacterial infections as it is a leading cause of sexually transmitted infection and blindness worldwide [44, 45]. In this point of view, it may be hypothesized that their stability as well-adapted pathogens in static environments for a long period have made them to keep gene transfers at a minimal level. In addition, the absence of *C*. *trachomatis* infecting phages detected may have impacts on the low ratio of HGT [11].

**Fig 4:**
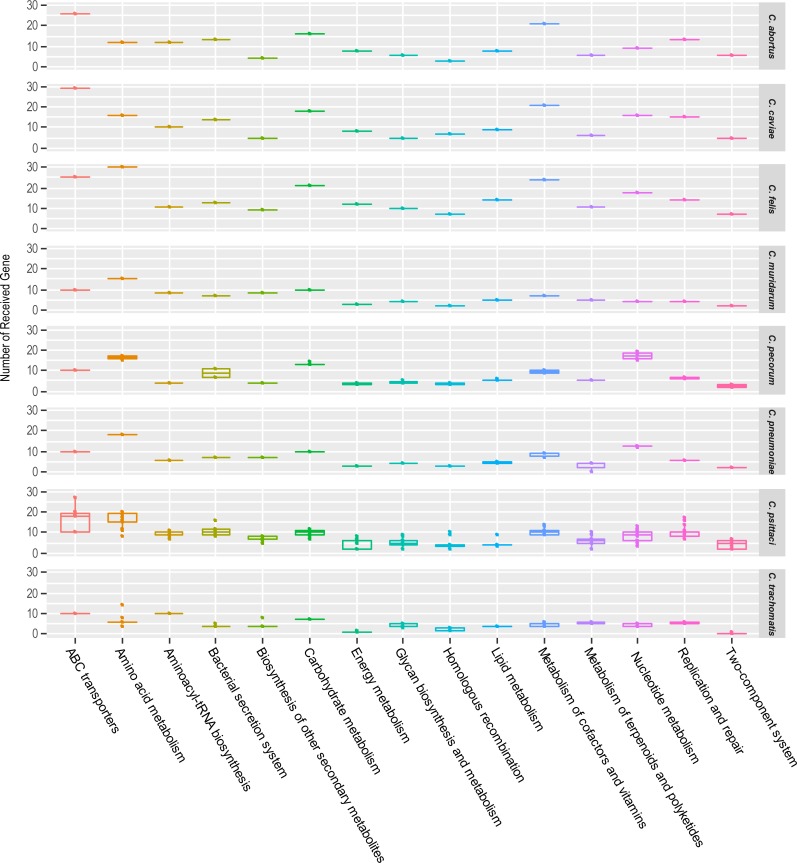
KEGG pathways of transferred genes of each *Chlamydia* species. The figure displays variation in the number of transferred gene sets across KEGG Pathways. The Boxplots represent the number of genes associated in corresponding KEGG Pathway transferred to each *Chlamydia* species. Each row denote 8 different *Chlamydia* species used in this study. The y-axis indicates the number of genes transferred counted.

**Fig 5:**
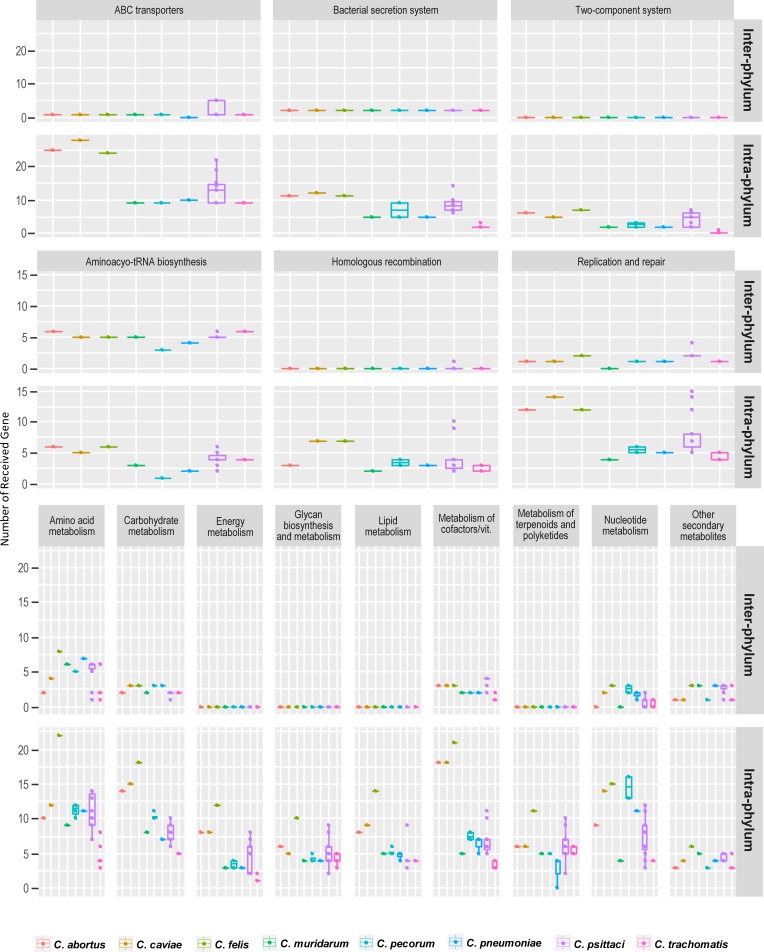
Distribution of number of the inter- and intra-phylum transferred genes across pathway. The figure displays variation in the number of transferred gene sets between inter-phylum HGT and intra-phylum HGT. The Boxplots show the number of genes transferred to each *Chlamydia* species distributed across KEGG Pathways. Each row denotes inter- and intra-phylum HGT genes. The y-axis indicates the number of received genes to *Chlamydia* species counted.

KEGG analysis of transferred gene families was performed to investigate pathways that might play important roles in *Chlamydia* and test whether transferred gene sets of each lineage are varied across functional pathways. In our analysis, variation in the number of transferred gene sets between lineages was quite similar across pathways, in most lineages, carbohydrate, nucleotide, and cofactor metabolism exchanged most abundantly (Fig 4). In previous study, frequent exchange of genes in carbohydrate transport and metabolism among animal associated organisms was observed [46]. However, we found that the effect of intra-phylum transfer and inter-phylum transfer on each pathway were different (Fig 5). For instance, genes associated in aminoacyl-tRNA biosynthesis pathway were horizontally acquired mainly from non-chlamydial species, and interestingly, HGT of the genes related to type III secretion system (T3SS) almost exclusively occurred at intra-phylum level (Fig 5). The varying effect on the functional categories and species suggest some pathways may accept the introducing of new genes from different phylum better than other pathways. Not all genes are permissive to interspecific gene transfer. Genes which encode proteins making up large complexes or genes whose products interact with other particles a lot display less preference towards HGT [47]. In addition, deleterious interaction between native and acquired proteins may be also important barriers [47, 48]. Barriers between certain species have been recognized as well for many bacterial and archaeal species [49].

### HGT of virulence-related genes of *Chlamydia*

Bacterial evolution is largely dependent on the ability to adapt and colonize specific niches. Gene transfers of virulence-related genes may affect the pathogenesis of specific *Chlamydia* strains. Therefore, it is important to identify these events to expand our understanding of speciation event and strain emergence. Although *Chlamydia* have very conserved genomes as a result of genome reduction imposed by their intracellular lifestyle [42], there is a region of hotspot for genome variation which is termed as “Plasticity zone,” and the region encodes virulence factors including membrane attack complex/perforin protein (MACPF), cytotoxin, and genes related to important biosynthesis and salvage pathways [50]. Presence or absence of these genes in the region are known to confer different niche-specificity to the organisms. Based on our work, there appeared to be several events of HGT in the plasticity zone occurring within *Chlamydia* genus. Since *Chlamydia* interact frequently with membranes during their infection, the role of MACPF is important [51]. We found the gene transfer of MACPF from *C*. *abortus* S26/3 to *C*. *felis* Fe/C-56 (SV: 1.0) and to *C*.*psittaci* M56 (SV: 1.0) separately. The tryptophan biosynthesis pathway is necessary for survival of *Chlamydia* since host restricts chlamydial growth by degrading tryptophan as a defense mechanism [52]. Each *Chlamydia* species possesses different level of functional gene sets of tryptophan biosynthesis pathway [53]. Among the genes associated in this pathway, only *TyrP* gene encoding tyrosine/tryptophan transport protein was transferred within *Chlamydia* genus (from *C*. *felis* Fe/C-56 to *C*. *caviae* GPIC; SV: 1.0). However, phylogenetic incongruence suggested that *TrpC* of *C*. *pecorum* E58, *C*. *felis* Fe/C-56, and *C*. *caviae* GPIC were might have been transferred from *Coxiella burnetti* dugway 5J108-111 strain, another obligate intracellular pathogen of humans and animals (SV: 1.0). *TrpC* encodes indole-3-glycerol phosphate synthase which is necessary in the fourth step of tryptophan biosynthesis pathway. It was previously proposed that *Coxiella burn*etti acquired trp operon from *simkania negevensis*, a bacterium belonging to the *Chlamydiae* phylum [54]. Putative multiple intra-genus gene transfers were also observed in adenosine deaminase (*Add*) associated in purine ribonucleotide biosynthesis pathway (S1 Fig), although no signs of HGT were detected in *GuaAB*. Presence of tox/adhesion may affect *Chlamydia* pathogenesis and host-range. These loci present only in *C*. *trachomatis*, *C*. *muridarum*, *C*. *pecorum*, *C*. *psittaci*, *C*. *caviae*, and *C*. *felis* species. To determine whether strains possessing the locus have gained the gene recently or whether these genes are lost by strains that do not is important [14]. According to our analysis, HGT of tox/adhesion had occurred from *Escherichia coli* and *Citrobacter rodentinum* to *C*. *caviae* GPIC (SV: 1.0).

*Chlamydia* displays a unique biphasic developmental cycle. At all stages of infection, interactions between the *Chlamydia* and its host are essential, and they translocate different types of virulence effector proteins into host cytoplasm which are used for manipulation of host cellular functions [55]. Like virulence genes in the plasticity zone, T3SS genes seemed to be mostly transferred within *Chlamydia*. Among the genes encoding structural components of the T3SS apparatus, there were evidences for intra-genus HGT in 5 genes (*SctW*, *SctS*, *SctR*, *SctF*, *and SctP*). Among the effector proteins that are used for manipulation of host cell immune response, *EEA1*, *Cap1*, *CPAF*, *Tsp*, and *pGP6-D* appear to have histories of HGT only with other *Chlamydia*.

HGT among *Chlamydiaceae* has been featured in many comparative genomics studies in recent few years [13, 14, 56]. We have discovered, using a tree reconciliation method, exchanges of virulence related genes are mainly occurred within *Chlamydia* genus. It suggests that intra-genus HGT may have been a major mechanism for the acquisition of determining factors of infection in *Chlamydia*. This discovery reflect that virulence related genes circulate among *Chlamydia* which may facilitate speciation event and new strain emergence. In microbial pathogens, virulence genes are particularly important determinants of host and tissue range [57], and transfer of those genes may provide a fitness benefit to the recipient. Gene acquisition from closely related species which have already adapted to meet the particular requirements of similar niche would confer even more advantages for adaptation. It will be informative to see if intra-genus HGT is a general mechanism for the acquisition of such factors also in other obligate intracellular pathogenesis. In this study, we uncovered several features of HGT acting on *Chlamydia* genome evolution and proposed an expansion of current understanding of *Chlamydia* ecology. More insights into HGT mechanism of *Chlamydia* will come from future laboratory experiments.

## Supporting information

S1 Fig Example of phylogenetic incongruence of adenosine deaminase (*Add*) gene. (A) is the species tree of *Add* gene. (B) is the gene tree of *Add* gene. Phylogenetic difference between the gene tree and the species tree indicate that *Add* gene inferred to have undergone one or more HGT events. The tree reconciliation method detected 3 putative HGT events in *Chlamydia*. The species highlighted with color red indicate the organisms participated in the events. Arrows depict the HGTs between the organisms. Support values are shown near to the corresponding HGTs which indicated by arrows.
(EPS)

S1 Table The information of Chlamydia strains used in this study. (XLSX)
